# Detection of Severe Acute Respiratory Syndrome Coronavirus 2 RNA on Surfaces in Quarantine Rooms

**DOI:** 10.3201/eid2609.201435

**Published:** 2020-09

**Authors:** Fa-Chun Jiang, Xiao-Lin Jiang, Zhao-Guo Wang, Zhao-Hai Meng, Shou-Feng Shao, Benjamin D. Anderson, Mai-Juan Ma

**Affiliations:** Qingdao Municipal Center for Disease Control and Prevention, Qingdao, China (F.-C. Jiang, Z.-G. Wang, Z.-H. Meng, S.-F. Shao);; Shandong Provincial Center for Disease Control and Prevention, Jinan, China (X.-L. Jiang);; Global Health Research Center, Duke Kunshan University, Kunshan, China (B.D. Anderson);; State Key Laboratory of Pathogen and Biosecurity, Beijing Institute of Microbiology and Epidemiology, Beijing, China (M.-J. Ma)

**Keywords:** Coronavirus diseases, 2019 novel coronavirus disease, COVID-19, SARS-CoV-2, severe acute respiratory syndrome coronavirus 2, respiratory diseases, zoonoses, viruses, pneumonia, hospitalization, RNA, environment, surface, transmission, hygiene, China

## Abstract

We investigated severe acute respiratory syndrome coronavirus 2 (SARS-CoV-2) environmental contamination in 2 rooms of a quarantine hotel after 2 presymptomatic persons who stayed there were laboratory-confirmed as having coronavirus disease. We detected SARS-CoV-2 RNA on 8 (36%) of 22 surfaces, as well as on the pillow cover, sheet, and duvet cover.

Severe acute respiratory syndrome coronavirus 2 (SARS-CoV-2) has rapidly spread globally and, as of May 2, 2020, had caused >3 million confirmed coronavirus disease cases (*1*). Although SARS-CoV-2 transmission through respiratory droplets and direct contact is clear, the potential for transmission through contact with surfaces or objects contaminated with SARS-CoV-2 is poorly understood (*2*). The virus can be detected on various surfaces in the contaminated environment from symptomatic and paucisymptomatic patients (*3*,*4*). Moreover, we recently reported detection of SARS-CoV-2 RNA on environmental surfaces of a symptomatic patient’s household (*5*). Because SARS-CoV-2 remains viable and infectious from hours to days on surfaces (*6*,*7*), contact with a contaminated surface potentially could be a medium for virus transmission. In addition, high viral load in throat swab specimens at symptom onset (*8*,*9*) and peak infectiousness at 0–2 days for presymptomatic patients (*8*) suggest that presymptomatic patients may easily contaminate the environment. However, data are limited on environmental contamination of SARS-CoV-2 by patients who may be presymptomatic. Therefore, to test this hypothesis, we examined the presence of SARS-CoV-2 RNA in collected environmental surface swab specimens from 2 rooms of a centralized quarantine hotel where 2 presymptomatic patients had stayed.

## The Study

Two Chinese students studying overseas returned to China on March 19 (patient A) and March 20 (patient B), 2020 (Table 1). On the day of their arrival in China, neither had fever or clinical symptoms, and they were transferred to a hotel for a 14-day quarantine. They had normal body temperatures (patient A, 36.3°C; patient B, 36.5°C) and no symptoms when they checked into the hotel. During the quarantine period, local medical staff were to monitor their body temperature and symptoms each morning and afternoon. On the morning of the second day of quarantine, they had no fever (patient A, 36.2°C; patient B, 36.7°C) or symptoms. At the same time their temperatures were taken, throat swab samples were collected; both tested positive for SARS-CoV-2 RNA by real-time reverse transcription PCR (rRT-PCR). The students were transferred to a local hospital for treatment. At admission, they remained presymptomatic, but nasopharyngeal swab, sputum, and fecal samples were positive for SARS-CoV-2 RNA with high viral loads (Table 1). In patient A, fever (37.5°C) and cough developed on day 2 of hospitalization, but his chest computed tomography images showed no significant abnormality during hospitalization. In patient B, fever (37.9°C) and cough developed on day 6 of hospitalization, and her computed tomography images showed ground-glass opacities.

**Table 1:** Timeline from return to China by 2 persons with presymptomatic SARS-CoV-2 infection to results of environmental sampling of their rooms at a centralized quarantine hotel, 2020*

Characteristic	Patient A	Patient B
Date returned to China	19 Mar	20 Mar
Date entered quarantine	19 Mar	21 Mar
Date of SARS-CoV-2 RNA–positive detection	20 Mar	22 Mar
Date of environmental surface sampling	20 Mar	22 Mar
Symptoms at hospital admission	None	None
C_t_ for clinical samples at admission†		
Nasopharyngeal swab	24.72	27.87
Sputum	28.61	23.23
Fecal swab	33.12	NA
Symptoms during hospitalization	Fever, chills, cough	Fever, cough, sputum, sore throat
Disease severity	Mild	Moderate

*C_t_, cycle threshold; NA, not available; rRT-PCR, real-time reverse transcription PCR; SARS-CoV-2, severe acute respiratory syndrome coronavirus 2. †A lower C_t_ indicates a higher viral load. Only C_t_ values for open reading frame1ab (*ORF*ab1) gene of SARS-CoV-2 were provided in the table because the Ct values for nucleoprotein were similar to values of *ORF*ab1.

Approximately 3 hours after the 2 patients were identified as positive for SARS-CoV-2 RNA, we sampled the environmental surfaces of the 2 rooms in the centralized quarantine hotel in which they had stayed. Because of the SARS-CoV-2 outbreak in China, the hotel had been closed during January 24–March 18, 2020. Therefore, only these 2 persons had stayed in the rooms. We used a sterile polyester-tipped applicator, premoistened in viral transport medium, to sample the surfaces of the door handle, light switch, faucet handle, thermometer, television remote, pillow cover, duvet cover, sheet, towel, bathroom door handle, and toilet seat and flushing button. We also collected control swab samples from 1 unoccupied room. We collected each sample by swabbing each individual surface. We tested the samples with an rRT-PCR test kit (DAAN GENE Ltd, http://www.daangene.com) targeting the open reading frame 1ab (*ORF*1ab) and *N* genes of SARS-CoV-2. We interpreted cycle threshold (C_t_) <40 as positive for SARS-CoV-2 RNA and C_t_ >40 as negative.

We collected a total of 22 samples from the 2 rooms of the quarantine hotel (Table 2). Eight (36%) samples were positive for SARS-CoV-2 RNA. C_t_ values ranged from 28.75 to 37.59 (median 35.64). Six (55%) of 11 samples collected from the room of patient A were positive for SARS-CoV-2 RNA. Surface samples collected from the sheet, duvet cover, pillow cover, and towel tested positive for SARS-CoV-2 RNA, and surface samples collected from the pillow cover and sheet had a high viral load; C_t_ for *ORF*1ab gene from the pillow cover was 28.97 and from the sheet, 30.58. Moreover, the C_t_ values of these 2 samples correlated with those of patient A’s nasopharyngeal (24.73) and fecal (33.12) swab samples at hospital admission. One surface sample from the faucet in patient B’s room was positive for SARS-CoV-2 RNA; the C_t_ was 28.75 for the *ORF*1ab gene. Again, we detected SARS-CoV-2 RNA from the surface samples of the pillow cover; C_t_ was 34.57. All control swab samples were negative for SARS-CoV-2 RNA.

**Table 2:** Results of environmental sampling of 2 rooms at a centralized quarantine hotel occupied by 2 presymptomatic SARS-CoV-2–infected patients, China, 2020*

Environmental source†	Values‡	
	Patient A’s room	Patient B’s room
Door handle	0/1	0/1
Light switch	1/1 (37.59)	0/1
Faucet	0/1	1/1 (28.75)
Bathroom door handle	1/1 (36.02)	0/1
Toilet seat, flush handle	0/1	0/1
Thermometer	0/1	0/1
TV remote	0/1	0/1
Pillow cover	1/1 (28.98)	1/1 (34.57)
Duvet cover	1/1 (35.64)	0/1
Sheet	1/1 (30.58)	0/1
Towel	1/1 (36.98)	0/1
Total, no. (%)	6/11 (54.5)	2/11 (18.2)

*Values are no. positive/total (C_t_) except as indicated. C_t_, cycle threshold; rRT-PCR, real-time reverse transcription PCR; SARS-CoV-2, severe acute respiratory syndrome coronavirus 2. †One swab was taken from each site except the light switch, from which 2 swabs were taken. ‡All samples taken from patients A and B after disinfection were negative and not included in this table. A lower C_t_ indicates a higher viral load. Only C_t_ values for open reading frame 1ab (ORFab1) gene of SARS-CoV-2 were provided because the C_t_ values for nucleoprotein were similar to those of *ORF*ab1.

## Conclusions

Our study demonstrates extensive environmental contamination of SARS-CoV-2 RNA in a relatively short time (<24 hours) in occupied rooms of 2 persons who were presymptomatic. We also detected SARS-CoV-2 RNA in the surface swab samples of the pillow cover, duvet cover, and sheet.

Evidence for SARS-CoV-2 transmission by indirect contact was identified in a cluster of infections at a shopping mall in China (*10*). However, no clear evidence of infection caused by contact with the contaminated environment was found. SARS-CoV-2 RNA has been detected on environmental surfaces in isolation rooms where the symptomatic or paucisymptomatic patients stayed for several days (*3*–*5*). In our study, we demonstrate high viral load shedding in presymptomatic patients, which is consistent with previous studies (*8*,*9*), providing further evidence for the presymptomatic transmission of the virus (*5*,*11*–*15*). In addition, presymptomatic patients with high viral load shedding can easily contaminate the environment in a short period.

Our results also indicate a higher viral load detected after prolonged contact with sheets and pillow covers than with intermittent contact with the door handle and light switch. The detection of SARS-CoV-2 RNA in the surface samples of the sheet, duvet cover, and pillow cover highlights the importance of proper handling procedures when changing or laundering used linens of SARS-CoV-2 patients. Thus, to minimize the possibility of dispersing virus through the air, we recommend that used linens not be shaken upon removal and that laundered items be thoroughly cleaned and dried to prevent additional spread.

The absence of viral isolation in our investigation was an obstacle to demonstrating the infectivity of the virus, but SARS-CoV-2 has been reported to remain viable on surfaces of plastic and stainless steel for up to 4–7 days (*6*,*7*) and 1 day for treated cloth (*7*). In summary, our study demonstrates that presymptomatic patients have high viral load shedding and can easily contaminate environments. Our data also reaffirm the potential role of surface contamination in the transmission of SARS-CoV-2 and the importance of strict surface hygiene practices, including regarding linens of SARS-CoV-2 patients.
